# Distinct iron isotopic signatures and supply from marine sediment dissolution

**DOI:** 10.1038/ncomms3143

**Published:** 2013-07-19

**Authors:** William B. Homoky, Seth G. John, Tim M. Conway, Rachel A. Mills

**Affiliations:** 1 Ocean and Earth Science, University of Southampton, National Oceanography Centre, European Way, Southampton SO14 3ZH, UK; 2 Department of Earth and Ocean Sciences, University of South Carolina, Columbia, South Carolina 29208, USA

## Abstract

Oceanic iron inputs must be traced and quantified to learn how they affect primary productivity and climate. Chemical reduction of iron in continental margin sediments provides a substantial dissolved flux to the oceans, which is isotopically lighter than the crust, and so may be distinguished in seawater from other sources, such as wind-blown dust. However, heavy iron isotopes measured in seawater have recently led to the proposition of another source of dissolved iron from ‘non-reductive’ dissolution of continental margins. Here we present the first pore water iron isotope data from a passive-tectonic and semi-arid ocean margin (South Africa), which reveals a smaller and isotopically heavier flux of dissolved iron to seawater than active-tectonic and dysoxic continental margins. These data provide *in situ* evidence of non-reductive iron dissolution from a continental margin, and further show that geological and hydro-climatic factors may affect the amount and isotopic composition of iron entering the ocean.

## Abstract (web summary)

The dissolution of iron from sediments along ocean margins may stimulate photosynthesis and moderate global climate. This study shows how margin sediments supply iron in varying amounts between regions, and by distinct mechanisms, which may be due to geological characteristics and hydrological controls on land.

## Graphical abstract

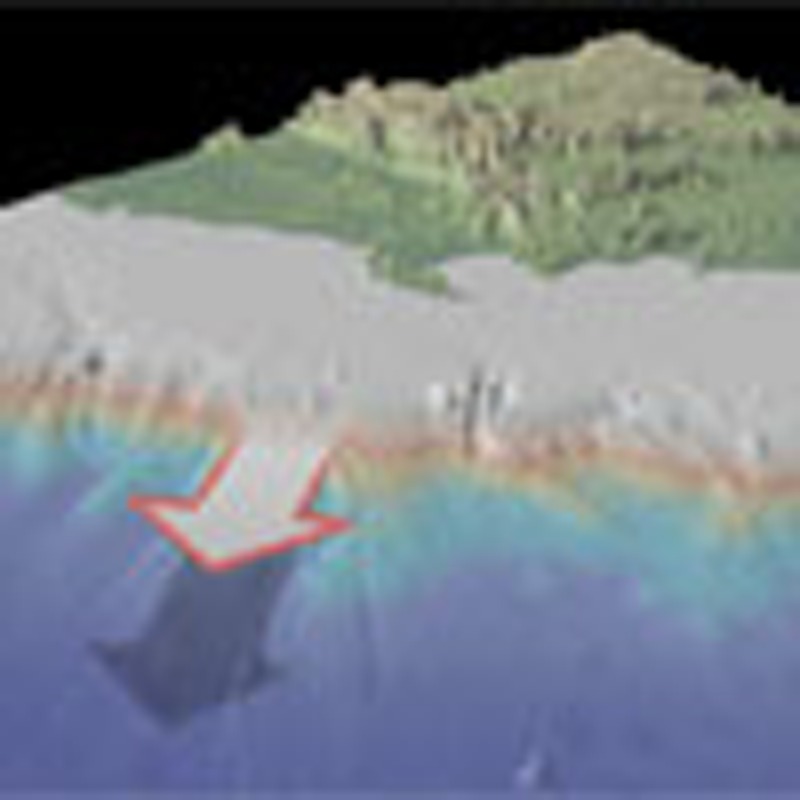

Iron (Fe) inputs to the surface ocean may stimulate photosynthesis and have an impact on the uptake of carbon dioxide in the ocean on glacial to inter-glacial timescales of climate change^1^. Global ocean reservoir-flux models^2^ indicate that 90% of Fe used by marine phytoplankton in the present day surface ocean is supplied from the deep water below, but the sources of dissolved Fe to this deep water are still poorly constrained. Therefore, quantifying and tracking iron supplied to the ocean will provide key information to resolve climate models and sensitivity to the Fe cycle^3^,^4^.

Measurable differences in the isotopic composition of Fe between various sources to the ocean have prompted widespread interest in seawater Fe isotope determintions^5^,^6^,^7^, which can potentially be used to track Fe inputs and assess the relative importance of different sources of dissolved Fe to the oceanic reservoir. Microbial sediment respiration supports a major flux of dissolved and isotopically light Fe to the global ocean^8^,^9^,^10^, by catalysing the reductive dissolution (RD) of Fe oxyhydroxide minerals during organic matter decomposition^11^. Reduction of Fe oxyhydroxide enriches soluble Fe(II)_(aq)_ in sediment pore water, which diffuses into bottom water when the oxygenated layer of surface sediment is adequately shallow^9^,^12^, most notably from oxygen-deficient continental margins^8^,^9^,^10^. Benthic fluxes of Fe are mixed in bottom waters and can be transported to open ocean and surface waters^13^,^14^, where Fe may control the efficacy of the biological carbon pump^15^,^16^.

Dissolved Fe(II)_(aq)_ produced by RD initially has δ^56^Fe values 0.5–2.0‰ lighter than the original substrates^17^, and at isotopic equilibrium, experiments show δ^56^Fe(II)_(aq)_ is −1.05 to −3.99‰ relative to the common reactive Fe oxides haematite^17^, goethite^18^ and ferrihydrite^17^,^19^,^20^. Similar light δ^56^Fe values (−1.82 to −3.45‰) have been observed in both the pore waters^21^,^22^,^23^ and overlying seawater^9^,^24^ of river-dominated and dysoxic margins, and light Fe isotopic compositions are recorded in ocean basin sediments coeval with past episodes of ocean oxygen deficiency, consistent with seawater transport of light Fe from ferruginous shelf sediments to ocean basins^25^. Thus, benthic fluxes of isotopically light Fe appear to be distinguishable from other sources of Fe to the ocean, such as atmospheric dust dissolution (δ^56^Fe=+0.13±0.18‰)^26^ and river discharge (δ^56^Fe=+0.14±0.28‰)^27^.

Paradoxically, however, equatorial Pacific seawater originating from the continental margin of New Guinea contains elevated Fe concentrations with heavy Fe isotopic compositions (δ^56^Fe=+0.37±0.15‰)^28^. These and other seawater isotope measurements have led to the proposition of an additional ‘non-reductive dissolution’ (NRD) mechanism for Fe^28^,^29^, albeit with existing Fe isotope evidence from continental margin sediments indicating otherwise^9^,^24^. These findings coincide with a growing need to evaluate the geographical variability of benthic Fe fluxes to effectively model carbon cycling in the ocean^3^,^4^, where models presently rely on global extrapolations from potentially unrepresentative regions.

Here we characterise the pore water isotopic composition and corresponding flux of dissolved Fe from the Cape margin, South Africa—a semi-arid passive margin derived from deeply weathered saprolite soils and surrounded by oxygenated South Atlantic seawater. These sites are distinct from most previous sites of benthic Fe flux investigation, which have focused on active margins next to areas of rapid uplift with oxygen-deficient shelf waters (Fig. 1). This study reveals that the amount of dissolved Fe released from the Cape margin is less than predicted by benthic Fe flux relationships^8^ widely used to model ocean Fe–CO_2_ interaction^3^,^4^. We report solid-phase compositional data that suggests that the small pore water Fe flux reflects geological and hydro-climatic influences on reactive Fe substrate delivery to the shelf. Isotopically heavy Fe present in ‘oxidizing’ pore waters of the Cape margin—a zone previously beyond analytical resolution—provides *in situ* evidence for the role of ‘NRD’ of Fe proposed by Radic *et al.*^28^ These discoveries have implications for past and present oceanic Fe cycles and the parameterization of ocean biogeochemical models.

## Results

### Geological setting

**Figure 1:**
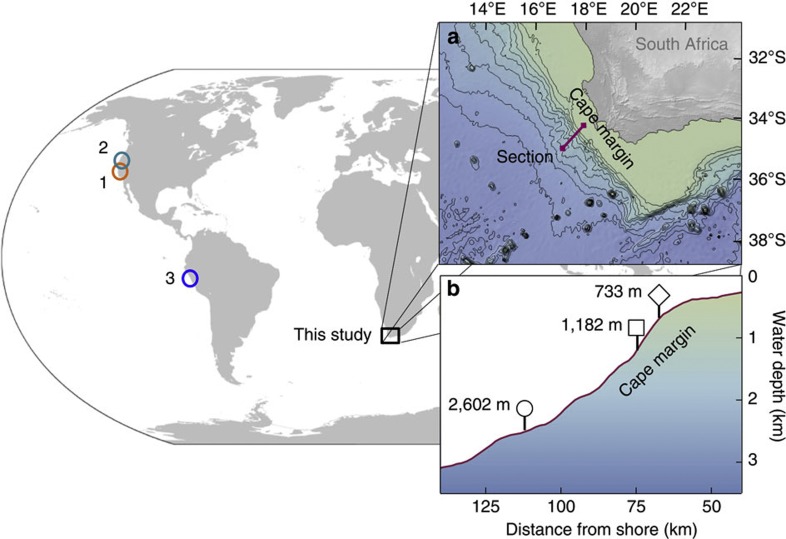
Location of benthic Fe flux determinations from ocean margins. The inset map (**a**) and corresponding cross-section (**b**) show the location of Cape margin sample sites (733 m, 1,182 m and 2,602 m) and their corresponding data markers (diamond, square and circle) used in accompanying figures. Models of ocean productivity and C-export^3^,^4^ assume that bio-essential Fe supplied from ocean margins corresponds to previous flux constraints from dysoxic borderland basins of Southern California (1)^8^,^9^,^44^, and supported by studies of seasonally dysoxic river-fed margins of California and Oregon (2)^9^,^12^ and the Peruvian margin oxygen-minimum zone (3)^10^. The Cape margin sites reveal a pronounced variability to the amount and isotopic composition of Fe that may be supplied from ocean margins between regions, which is not yet well accounted for by global ocean biogeochemical models.

Cape margin sediments originate from Palaeozoic sedimentary sequences of the Cape Supergroup (quartz arenites, subgreywackes, shales and sandstones) that were formed by weathering of ancient Kalahari, Patagonia and Río de la Plata Cratons and deposited along rifted margins of Southern Gondowanda ~480–300 Ma 30(ref. 30). The modern Berg and Olifants Rivers drain the Cape Supergroup and outflow 100–200 km north of our study area, slowly delivering mature siliciclastic material to the Cape shelf (<3 cm kyr^−1^) (ref. 31), where shelf deposits have been further re-worked by Neogene sea-level fluctuations^32^. Consequently, winnowed glauconitic sands and quartzose muddy sands occupy the outer shelf and upper-shelf slope^31^, from where we collected surface sediments from three sites at 733, 1,182 and 2,602 m water depth (Fig. 1). South Atlantic water masses intersecting the sample sites are replete with oxygen, with a small oxygen minimum (174 μmol l^−1^) associated with Upper Circumpolar Deep Water intersecting the 1,182 m study site.

### Cape margin sediment composition

**Figure 2:**
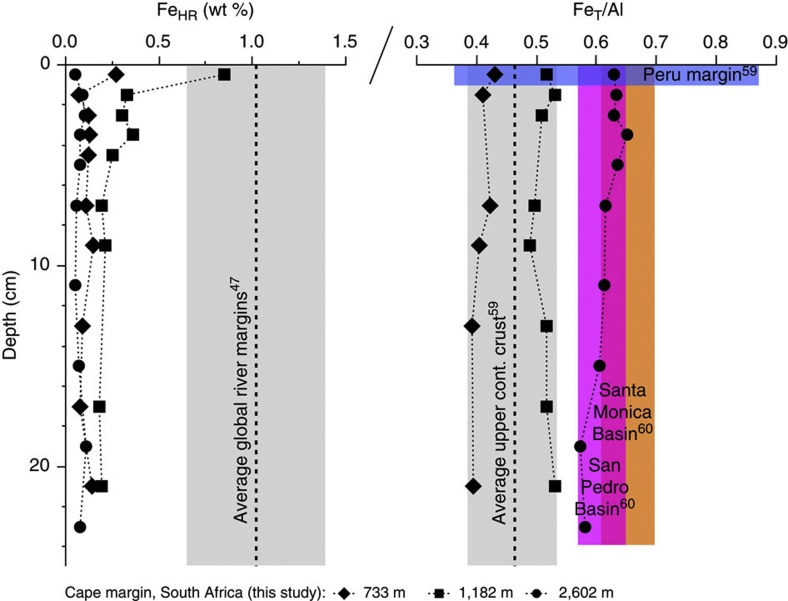
Fe abundance in Cape margin sediments. The amount of highly reactive Fe (Fe_HR_) and the proportion of total Fe to Al is shown for the Cape margin—a semi-arid passive-tectonic margin—compared with global averages and sites previously used to characterize the benthic Fe flux to the oceans. Grey bars represent the two times the s.d. of mean values from literature; coloured bars represent the range of abundances determined across corresponding sediment depths. Fe_HR_ is liberated by a Na-dithionite extraction^55^ and used here to show that the reducible Fe oxide reservoir in Cape margin sediments is depleted relative to the global average of river-dominated margins. Upper-shelf slope sites also contain crustal Fe/Al ratios, indicating little enrichment of authigenic Fe compared with sites used for previous benthic Fe flux determinations.

The presence of quartz, plagioclase and K-feldspars, calcite and clay fractions of illite and glauconite were confirmed by XRD and scanning electron microscopy (SEM) analyses. Iron was often associated with Ti and O as illmenite minerals, whereas Fe-S phases were not identified by XRD or energy-dispersive X-ray spectroscopy (EDS) point analysis. The Cape margin sediments contained substantially fewer reducible Fe oxide minerals (Fe_HR_) than have been observed in river-dominated margin sediments from around the world (Fig. 2; Table 1). Furthermore, total Fe in Cape margin sediments largely reflects the average crustal abundance in contrast to areas influenced by river discharge or dysoxic ocean waters that are typically enriched above crustal Fe concentrations and therefore have a more replete Fe reservoir for dissolution processes. Thus, Cape margin sediments provide a previously missing case study to link earth surface processes with the nature of seawater Fe supply from the continents.

### Cape margin pore water composition

**Figure 3:**
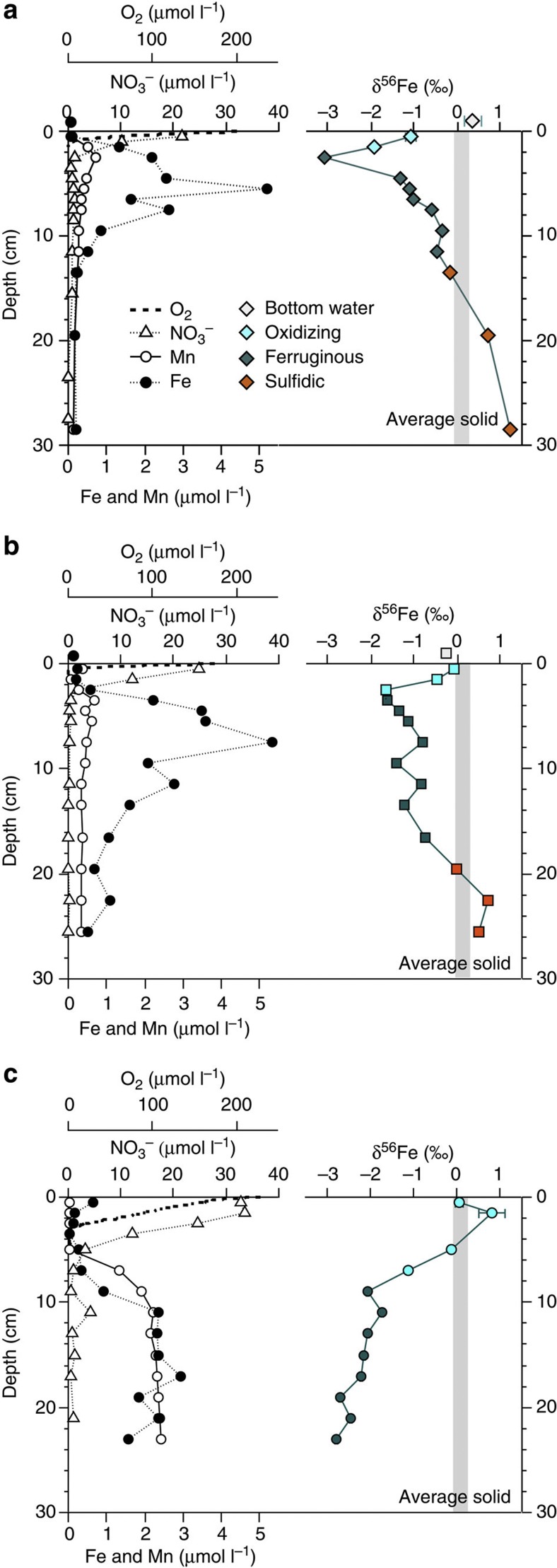
Pore water geochemical profiles for Cape margin. Down-core distribution of dissolved O_2_, NO_3_^−^, Mn and Fe in Cape margin sediment pore water from (**a**) 733 m, (**b**) 1,182 m and (**c**) 2,602 m water depths. Chemical zones are described with respect to dissolved Fe as ‘oxidizing’ (O_2_ and/or NO_3_^−^ in the pore water), ‘ferruginous’ or ‘sulphidic’ (Fe depletion consistent with SO_4_^−^ reduction). Corresponding pore water iron isotopic compositions express distinct behaviour across chemical zones, which are broadly reproduced at each site (see text for further details).

The upper-shelf slope of the Cape margin (733, 1,182 and 2,602 m water depth) contains sufficient organic carbon (means 2.2, 1.7 and 1.4 wt%; 0–20 cm) to consume dissolved O_2_ within just a few millimetres of the sediment–water interface (Fig. 3; Table 1). Subsequent down-core chemical zonations of dissolved NO_3_^−^, Mn and Fe in the pore water are consistent with control by organic matter decomposition (Fig. 3). Sub-surface dissolved Fe maxima (between 3 and 6 μmol l^−1^) are clearly identified in what have been termed ‘ferruginous’ zones between relatively ‘oxidizing’ (O_2_ and NO_3_^−^ in the pore water) and ‘sulphidic’ (Fe depletion consistent with SO_4_^−^ reduction^33^) zones. However, dissolved Fe values remain 10–100 times lower than pore waters from California^8^,^9^,^12^,^21^,^23^, Oregon^9^,^12^,^21^ and Peru^10^ margins, where previous benthic Fe flux investigations have been focused. The speciation of dissolved Fe in the sampled pore water is not known, but the near-surface abundance of electron acceptors means dissolved Fe in the oxidizing zone may be present as either Fe(II)_(aq)_ colloidal oxyhydroxides^34^ or organic complexes.

### Cape margin iron isotope signatures

We observe a wide range in the Fe isotopic composition of Cape margin pore waters (δ^56^Fe=−3.09 to +1.22‰ relative to IRMM-14), but with distinct and reproducible down-core behaviour between sulphidic, ferruginous and oxidizing zones in the sediments (Fig. 3; Table 1). We find δ^56^Fe of the bulk solid phase to be +0.08±0.2‰ (*n*=11), equal to the average weathering product of the continental crust (+0.09±0.7‰) described by previous workers^36^. Relatively heavy ‘sulphidic’ zone pore water Fe isotopes at depth in these sediments are consistent with control by low-solubility sulphide minerals observed elsewhere^21^, and supported by experimental studies of pyrite formation, in which pore water Fe(II)_(aq)_ may reach +1.3‰ when not in equilibrium with the solid phase^37^. We observe the lightest Fe isotopic compositions (−0.34 to −3.09‰) in the ferruginous zone at each study site, which is characteristic of microbially catalysed RD seen in marine sediments elsewhere^9^,^21^,^23^ and supported by incubation experiements^17^,^20^. However, the isotopic composition of Fe in the most oxidizing layer of surface sediments shows a systematic and previously unidentified transition towards heavier, near-crustal values at the sediment–water interface.

**Figure 4:**
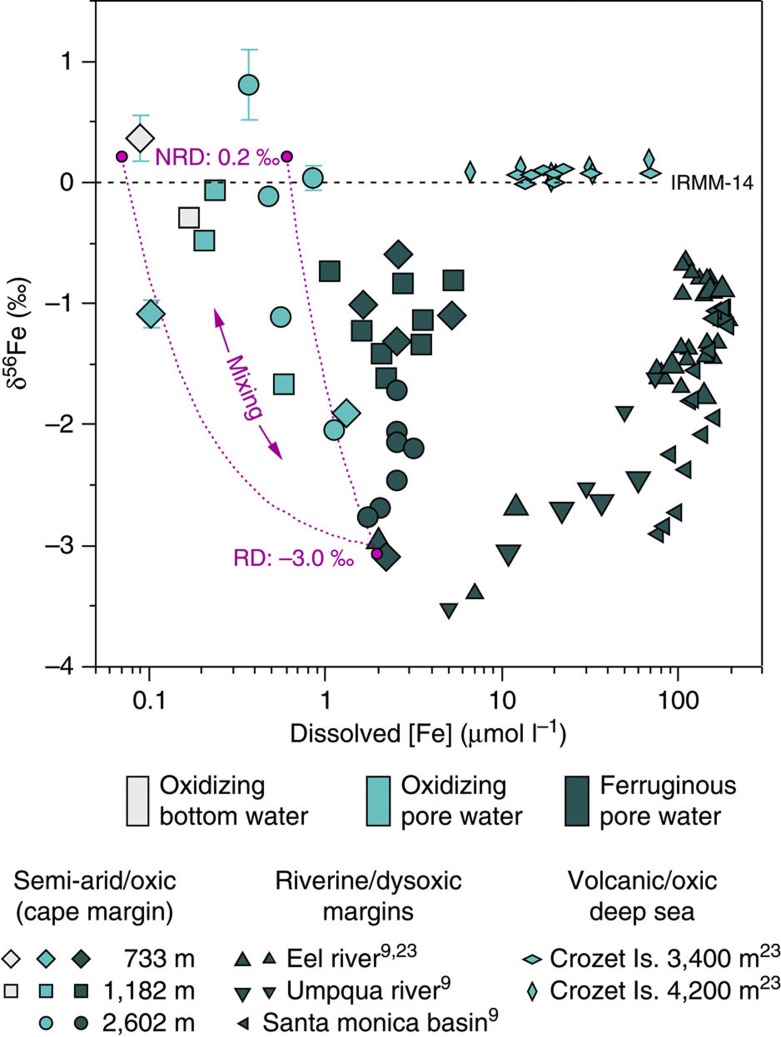
Benthic δ^56^Fe signatures of marine sediment dissolution. The log-normal scatter plot compares the concentration and isotopic composition of dissolved Fe observed between the oxidizing and ferruginous zones of pore waters from the semi-arid Cape margin (this study), river-dominated sites of California and Oregon margins, and volcanogenic deep-sea sediments of the Southern Ocean. The relatively low abundance of dissolved Fe on the Cape margin is attributed to the scarcity of reducible Fe oxides in these sediments. The isotopic composition of dissolved Fe in the ‘oxidizing’ pore waters of the Cape margin are best described by mixing between RD and NRD end-member Fe sources. The high abundance of Fe with NRD isotopic compositions in the volcanic and oxygenated deep sea is consistent with more rapid alteration of the primary volcanic minerals derived from the Crozet Island basalts^34^.

The pore water Fe isotopic concentration variations that we observe could not have been generated by oxidation or sorption of the reduced Fe pool, and nor could they be oxidation-related sampling artifacts. Precipitation of Fe(II)_(aq)_ by oxidation and/or sorption has a kinetic isotope effect that lowers the residual δ^56^Fe(II)_(aq)_^17^ (ref. 17). We hypothesize that the near-surface transition to heavier Fe isotopic compositions reflects mixing with a heavier end-member Fe input, which is only discernable because of the very low abundance of dissolved Fe supplied by RD at these sites compared with previous continental margin studies (Fig. 4).

Potential sources of heavy Fe isotopes include dissolution of sulphide, oxide and silicate minerals. The equilibrium isotopic fractionation between Fe(II)_(aq)_ and FeS_(s)_ has been experimentally determined (−0.32±0.29‰)^37^, but previous field observations of Fe(II)_(aq)_ in the presence of FeS_(s)_ have been restricted below the ferruginous zone^21^, where pore waters do not reflect equilibrium conditions^37^. In surface sediments, FeS_(s)_ is commonly unstable and readily forms Fe oxyhydroxide minerals^38^. Multiple solid-phase spectroscopic analyses did not identify any FeS or FeS_2_ minerals in the oxidizing surface layer of the Cape margin study sites. Therefore, any Fe-S minerals physically entrained in the surface layers have probably already contributed to the authigenic pool of reactive Fe oxide minerals.

Isotopically heavy Fe in pore water Fe could be attributed to an equilibrium isotopic effect during NRD of oxide and/or silicate weathering products on the Cape margin; for example, the isotopic composition of Fe in oxidizing pore waters is also heavy (+0.16±0.05‰)^23^ in deep-sea volcanogenic turbidites, where dissolved Fe is dominated by colloids formed by inorganic dissolution of volcanic minerals^34^. Other disparate lines of evidence exist for a common equilibrium isotopic effect; first, dissolved Fe released to seawater from atmospheric dust is heavy (δ^56^Fe of +0.13±0.18‰)^26^, and confirmed beneath Saharan dust plumes in the North Atlantic where elevated surface ocean Fe concentrations have a δ^56^Fe of +0.33±0.05‰^39^ (ref. 39); second, dissolved Fe in river water is heavy (δ^56^Fe of +0.14±0.28‰)^27^ and persevered through the estuarine mixing zone despite intense removal during flocculation, indicative of isotopic equilibration with suspended solids; and finally, dissolved Fe in New Guinea coastal waters influenced by sediment re-suspension has a δ^56^Fe of 0.37±0.15‰, 0.2‰ heavier than the suspended particles^28^. The remarkable consistency in dissolved Fe isotopic compositions across a diverse set of oxygenated sediment–seawater interactions is used here to predict the mean isotopic fingerprint of dissolved Fe released by NRD at our study sites (mean δ^56^Fe=+0.22±0.18‰), and we find this consistent with the predictions of Radic *et al.*^28^

We consider the amount of Fe in surface sediment pore waters that may originate from NRD on the Cape margin using the estimated end-member isotopic composition of dissolved Fe supplied by reductive and NRD processes. We derive isotope-mixing lines in Fig. 4 with a standard two-component mixing calculation, where the slope (*a*) and intercept (*b*) describe the relationship between δ^56^*Fe* and [Fe] in equation (1). Equations (2) and (3) define terms *a* and *b*, where the two end-member isotopic compositions and their respective concentrations in the pore water are set by reasoned constraints from the literature and values befitting to the Cape margin data set;


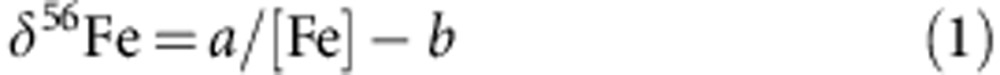



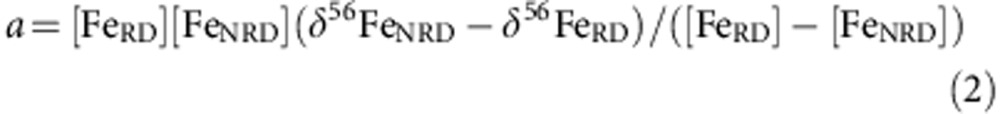



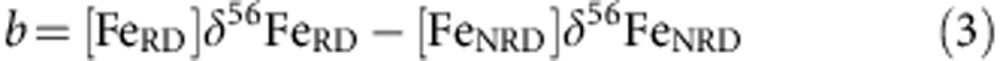


Fe supplied by RD (Fe_RD_) is defined as having δ^56^Fe_RD_=−3.0‰ (17,20), and a hypothetical concentration of 1.75 μmol l^−1^, whereas δ^56^*Fe*_NRD_=0.22±0.18‰. When [Fe_NRD_] is set between 0.07 and 0.6 μmol l^−1^ in the surface sediment (a majority of the observed pore water [Fe]), these parameters provide two mixing lines (Fig. 4), which approximate the observed relationship between δ^56^Fe and [Fe] between ferruginous and oxidizing zones on the Cape margin. Pore water Fe isotopic data and the implied mechanism of pore water Fe supply have implications for the isotopic composition and flux of Fe from the Cape margin, as well as the geographical distribution and isotopic diversity of Fe supply to the global ocean.

### Iron supply from the Cape margin

A one-dimensional diffusion-oxidation calculation using pore water Fe and O_2_ concentration data^40^ has previously been used to estimate the benthic flux of Fe from sites on the California and Oregon shelves, where it was validated by direct field comparison with *in situ* and *ex situ* incubation techniques^12^. We use the same approach to estimate the benthic flux of Fe to bottom waters from the Cape margin. The flux (mol cm^−2^ s^−1^) of Fe^2+^ (*J*) is described in equation (4), where *ϕ* is the sediment porosity, *L* is the thickness (cm) of the oxygenated layer of surface sediment and *C*_p_ is the concentration (g cm^−3^) of Fe^2+^ in the pore water beneath *L*.


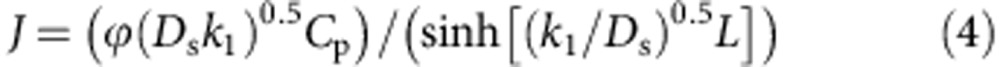


The rate constant for Fe^2+^ oxidation is described by *k*_1_ (s^−1^) in equation (5), as a function of bottom water O_2_ concentration (mol kg^−1^), pH and the value of *k* (3.54 × 10^−1^ kg mol^−1 ^s^−1^) derived from the relationships between temperature (6 °C) and salinity (34) with Fe^2+^ oxidation in seawater^41^.


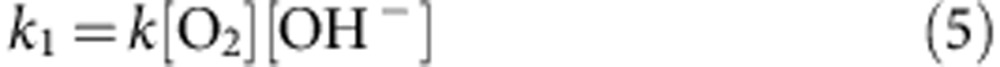


The diffusion coefficient^42^ of Fe^2+^ in muddy shelf sediment pore waters (cm^2^ s^−1^) is described in equation (6), as a function of *ϕ*, corrected for tortuosity, and temperature *T* (°C).


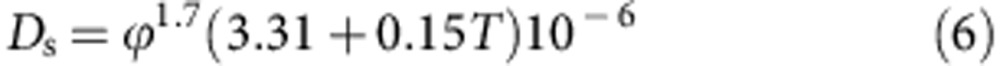


We use a *ϕ* value of 0.77 determined by Multi-Scan Core Logging of archived sediment cores at the National Oceanography Centre (NOC), and in the absence of direct determination, pore water pH is assumed to be 7.5±0.1 (ref. 12). Bottom water [O_2_] is derived from shipboard Winkler titrations of near-bottom water samples of 201 and 174 μmol l^−1^ at 733 and 1,182 m, respectively. Values of *L* are derived from O_2_ microsensor determinations from sites 733 and 1,182 m of 1.05 and 0.62 cm, respectively, and we use a depth-corrected pore water [Fe] beneath *L* to derive corresponding *C*_p_ values of 0.84 and 0.24 μmol l^−1^. The calculated fluxes of Fe to bottom water from the 733 and 1,182 m Cape margin sites are 0.11±0.13 and 0.23±0.17 μmol m^−2^ d^−1^, respectively.

**Figure 5:**
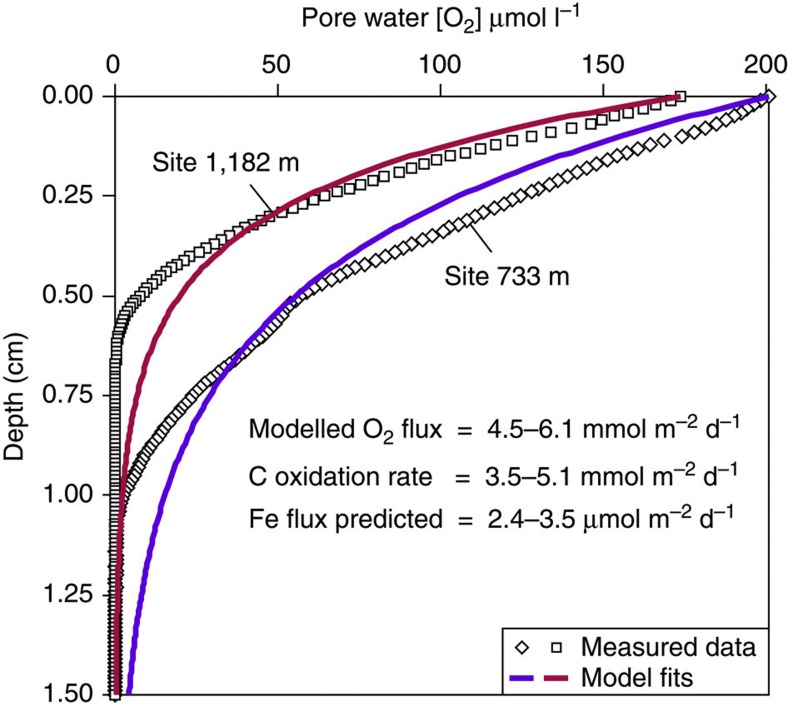
Benthic Fe flux prediction from C oxidation rates. Comparison of pore water O_2_ concentration versus depth profiles with model fits to data from steady-state pore water O_2_ consumption by organic C oxidation^44^. The corresponding rates of organic C oxidation are used to predict benthic fluxes of Fe from the Cape margin based on correlated C oxidation and Fe flux observations from the California margin^8^. However, despite comparable rates of organic C oxidation between these regions, this approach predicts benthic Fe fluxes higher than calculated from pore water Fe data in this study.

We find values of benthic Fe flux to be 2–4 orders of magnitude smaller than widely cited and most recent constraints from active-tectonic and dysoxic continental margins^8^,^9^,^10^,^43^. Furthermore, the flux of Fe is substantially lower than predicted by the application of the Elrod *et al.*^8^ relationship to organic C oxidation rate; Elrod *et al.*^8^ show that benthic Fe fluxes correlate with rates of organic C oxidation for numerous sites along the California margin, and the relationship is used to extrapolate benthic fluxes of Fe from organic C fluxes for ocean biogeochemical models^3^,^4^. Using a one-dimensional steady-state O_2_ diffusion-consumption model, we approximate the rate of organic C oxidation by fitting calculated outputs to the observed down-core profiles of O_2_ concentration determined by microsensors (Fig. 5). The approach follows Berner^44^, in which a single pool of reactive organic C is assumed to be the only mechanism of O_2_ utilization (for example Papadimitriou *et al.*^45^), and the influence of biophysical mixing, seasonal accumulation rates and porosity structure are ignored. The rates of organic C oxidation for sites 733 and 1,182 m (3.5 and 5.1 mmol m^−2^ d^−1^) are derived from the modelled flux of O_2_ and the stoichiometry of organic matter remineralization, and are equivalent to rates of organic matter decomposition from previous sites of benthic Fe flux determination^8^. Following Elrod *et al.*^8^, we predict a benthic Fe flux of 2.4–3.5 μmol m^−2^ d^−1^ from the Cape margin (Fig. 5), and find this an order of magnitude more than we calculate from pore water Fe data. Thus, Cape margin sediments appear to deviate from the relationship between sediment respiration and benthic Fe supply rates observed on North American river-dominated and dysoxic margins of the Pacific Ocean.

## Discussion

**Figure 6:**
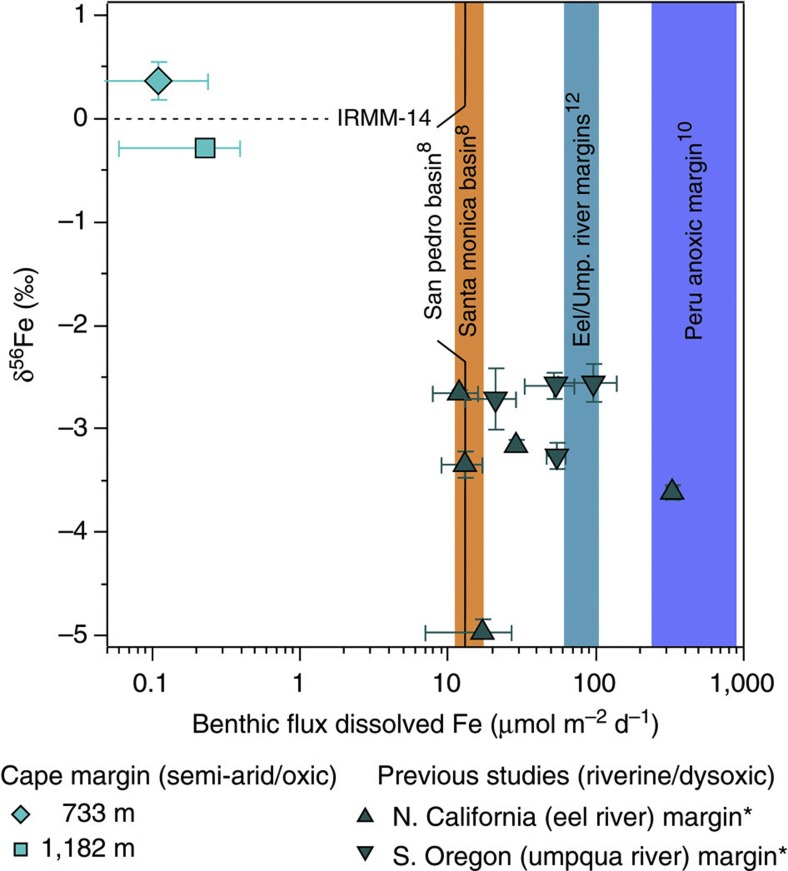
Benthic flux and isotopic composition of dissolved Fe. The benthic flux of dissolved Fe and its corresponding isotopic composition is shown for the semi-arid and passive-tectonic Cape margin of South Africa (this study) compared with previous studies of river-dominated, dysoxic and active-tectonic margin settings. Coloured bars correspond to the range of benthic Fe flux determinations where Fe isotopic composition was not also determined.

Cape margin sediments indicate that the supply of Fe to the southeast Atlantic Ocean is smaller and isotopically heavier than current models of Fe cycling would suggest^8^,^9^,^24^ (Fig. 6). Both of these findings have widespread implications for the marine Fe cycle. It appears that the relationship proposed by Elrod *et al.*^8^, which for a decade has provided the most widely used constraint on the benthic flux of Fe to the global oceans, may overestimate benthic Fe flux from margin sediments with lower reactive Fe inventories or less-effective mechanisms for Fe enrichment.

Continental margin sediments are a reservoir for reactive iron mineral substrates, which supply dissolved Fe to the oceans and thereby support ocean life. Globally, rivers provide three quarters of the particulate Fe content of continental margins^46^, where on average sediments comprise 1 wt% highly reactive Fe oxides (Fe_HR_)^47^. Tectonic uplift enhances sediment transport to the ocean from active orogenic belts due to many factors (fractured and brecciated rocks, over-steepened slopes and seismic and volcanic activity) in addition to elevation/relief^48^. Hydro-climatic conditions provide a second-order influence on sediment transport to the oceans^48^,^49^, where continental run-off intensity increases the Fe_HR_ enrichment of sediment carried by rivers^47^. For example, Indian margin sediments record monsoon intensity with enrichments of Fe_HR_ and total Fe (Fe_T_) to Al ratios nearly double the continental average on decadal timescales^50^,^51^. Therefore, tectonics and climate are effective ways to mediate the supply and enrichment of Fe_HR_ at ocean margins.

The Cape margin is depleted in Fe_HR_ (0.17 wt%, 0–20 cm) compared with the global average of continental margins^47^ (Fig. 2). Prolonged tectonic stability in this region and a semi-arid climate have probably contributed to the relatively slow rate of sediment accumulation^31^—perhaps allowing the rate of Fe_HR_ reduction and dissolution to meet or exceed the rate of Fe_HR_ supply and produce the low Fe_HR_ content observed. We consider the limited abundance of reactive Fe oxide minerals as the most likely means of restricting the flux of dissolved Fe to seawater in this region. Thus, shifting patterns in tectonic and hydro-climatic conditions might have influenced the Fe inventory of margin sediments in the past, with unknown impact on regional and global inputs of dissolved Fe to seawater.

The limited amount of RD on the Cape margin reveals the co-existence of a NRD process, releasing Fe in the oxidizing layer of surface sediments. The discovery is consistent with predictions based on seawater isotopic compositions of Fe and Nd measured elsewehere^28^,^52^. Dissolution rates may be slower than microbially catalysed RD, but the process could be widespread. Non-reductive Fe dissolution is likely to reflect physical and compositional variations in sediments influenced by geological provenance and weathering rates, as indicated by the high abundance of Fe in oxidizing volcanic sediments around the Crozet Islands (Fig. 4). Using a sediment respiration parameter to estimate Fe supply from marine sediments is unlikely to account for the distribution and magnitude of Fe released by NRD of marine sediment. Readily weathered volcanic and oxygenated sediments are prevalent across ocean basins^34^, and dilute sediment suspensions can be transported hundreds of kilometres offshore where they influence primary production^13^,^53^, so multiple mechanisms for Fe dissolution may have a far-reaching influence on seawater isotopic compositions and productivity.

Cape margin Fe isotopic data remain consistent with interpretations of isotopically light Fe in modern marine and ancient sediment records^25^,^54^ in which anoxic seawater is suggested to shuttle isotopically light Fe from ferruginous margin sediments to ocean basins. However, the measured or inferred absence of light Fe isotopic compositions in seawater would be a poor assessment of the oceanic Fe inventory and its impact on primary productivity, given that we now need to consider the variables of non-reductive Fe dissolution.

Cape margin sediments shed light on Fe exchange between a semi-arid tectonically passive continental margin and oxygenated ocean. The sediments host distinct mechanisms of Fe dissolution, resulting in a smaller, isotopically heavier input to seawater than predicted. Semi-arid passive margin environments are common, and their distribution varies over time, thus requiring appraisal when reconstructing past ocean conditions. In addition to the distribution of ocean anoxia, we predict that the proportion of dissolved Fe supplied to the ocean by reductive and non-reductive sediment dissolution will reflect patterns in continental weathering and transport to ocean margins. Slow rates of reactive Fe substrate delivery to ocean margins may limit the benthic flux of Fe by RD relative to organic C oxidation rates. In addition, young volcanic terrains that are easily weathered are likely to release greater amounts of Fe by NRD compared with the mature sediment lithologies of the Cape margin. Regional constraints on oceanic Fe supply are missing links for modelling the coupled ocean-atmosphere carbon cycle^3^,^4^. Therefore, evaluating Fe supply from additional and diverse ocean boundaries remains an essential goal for modelling climate.

## Methods

### Sediment and pore water sampling

A Bowers and Connelly Megacore and Box core sampled shallow (<0.4 m) sediment and pore water from three sites on the Cape margin (733, 1,182 and 2,602 m; Fig.1, Table 1) during the UK GEOTRACES A10 expedition from the RRS *Discovery* (D357) in October–November 2010. Sub-samples for oxygen profiling and pore water extraction were collected by polycarbonate push core, and shipboard processing was performed at ambient bottom water temperatures.

Rhizon samplers collected dissolved pore water and overlying seawater constituents for elemental analysis (filtration cut-off of 0.15 μm). Rhizon samplers (50 × 2.5 mm) were pre-soaked in 18.2 MΩ de-ionized water and inserted through pre-drilled holes in core tubes at 1–3 cm intervals down-core. A ‘BD Discardit’ 20 ml syringe (pre-cleaned: 72 h 10% Decon; 72 h 6 M HCl; 72 h 6 M HNO_3_; rinsed by 18.2 MΩ de-ionized water) and secured to each Rhizon by Luer-lock connection. A brace inserted between each syringe housing and plunger applied suction to Rhizons. The first 0.5 ml of sample was discarded. Rhizons drew ~6 ml of pore water (10–20 min) and were divided for macronutrient and metal analysis. The aliquot for metals was acidified (pH<2) directly through the syringe tip (6 μl of 6 M quartz-distilled (Q-)HCl per ml of pore water) and transferred to low-density polyethylene (LDPE) pots. Residual sediments were divided for elemental analyses by extruding and slicing cores with a Teflon sheet at 1–2 cm depth intervals. Sediments were later freeze-dried and homogenized by agate pestle and mortar.

### Sediment digestion and leaching procedures

Heated Aqua Regia and combined HF-HClO_4_ acid dissolved sediment samples following an established protocol at the NOC in Southampton^34^. Digested sediment residues were re-dissolved in 6 M HNO_3_ in preparation for analysis by Inductively Coupled Plasma-Mass Spectrometry (ICP-MS). Digestion of the certified standards SCO-1, SGR-1 (United States Geological Survey) and BCSS-1 (National Research Council Canada) allowed for the recovery of Fe, Mn and Al within consensus values (Table 2).

Highly reactive Fe (Fe_HR_) is operationally defined as Fe liberated by dissolution during a 2-h reaction with Na dithionite designed to target reducible Fe oxide phases (for example, ferrihydrite, lepidocrosite, goethite and haematite)^55^. A 10 ml aliquot of 50 g l^−1^ Na-dithionite solution buffered to pH 4.8 with 0.35 M acetic acid and 0.2 M Na citrate was reacted at room temperature with ~100 mg of dry sediment sample. The resultant solution was spun for 8 min by centrifuge at 9,000 *g*. Supernatant containing dissolved Fe was decanted into LDPE before analysis by ICP-MS.

### Element abundance by ICP-MS

A Perkin Elmer Element X2 ICP-MS measured the concentration of Fe and Mn in pore water, and Fe and Al in sediment solutions at the NOC. Sediment digests were diluted 1,000 times, and pore water samples 100 times, with a 0.48 M Q-HNO_3_ solution containing 2 ng g^−1^ Re, Rh and Sc and 5 ng g^−1^ Be as internal standards. External calibration standards were prepared from certified stock solutions. For dilute pore water analyses, standards were matrix matched with 1% seawater (NASS-5; National Research Council Canada), effectively adding a small known amount of Fe (35 pmol l^−1^) and Mn (160 pmol l^−1^) to the standards that is corrected for during external calibration.

Samples were introduced by an Elemental Scientific SC-4 DX Autosampler and PC^3^ Peltier cooled inlet system with integrated cyclonic spray chamber at 100 μl min^−1^. Masses ^56^Fe, ^55^Mn and ^27^Al were measured in medium resolution mode. Internal standards were monitored throughout and used to correct for the reduction in signal intensity over time. The accuracy of the method was verified by the intermittent analysis of blank-bracketed SLRS-5 within certified values (Table 2), with a relative s.d. <0.5%. The detection limits (3 s.d. of analytical blanks, *n*=11) for Fe, Mn and Al were 24, 4 and 14 nmol l^−1^, respectively. Mean procedural blanks (*n*=4) for pore water sampling and analyses were 85±3.5 nmol l^−1^ for Fe and below detection for Mn.

### Fe isotope determinations by Multi-Collector ICP-MS

The isotopic composition of Fe in pore waters and sediments was assessed using a modification of John and Adkins^7^. Sample solutions containing 17–115 ng of Fe were quantitatively spiked with a ^57^Fe–^58^Fe double spike^56^ using a spike:sample ratio of 2:1. Spike-sample mixtures were dried in Savillex PFA Teflon vials and re-dissolved with 5 M Q-HCl+0.001% v/v Fisher Scientific Optima. A 135 μl aliquot of acid-cleaned AG-MP1 anion exchange resin was used in LDPE columns (pre-cleaned: 72 h 10% v/v decon, 1 week 6 M HCl) for the separation of Fe from sample matrices. Resin-filled columns were rinsed with 2M Q-HNO_3_ and conditioned with 5 M Q-HCl+0.001% v/v H_2_O_2_ before loading spiked samples in 100 μl aliquots. Loaded columns were rinsed by 12 × 100 μl aliquots of 5 M Q-HCl+0.001% v/v H_2_O_2_. Fe was eluted by 800 μl of 1 M Q-HCl into Savillex PFA Teflon vials, dried and re-dissolved in 2 ml of 0.1 M Q-HNO_3_ before analysis by Multi-Collector (MC) ICP-MS. Column calibrations assessed procedural blanks (2.7±0.6 ng of Fe, *n*=2) and recovery of Fe (>95%). Calibrations also confirmed the effective separation of Fe from major salts (Ca) and interferences (^58^Ni and ^54^Cr).

A Thermo Scientific (Neptune) MC ICP-MS-measured Fe isotope ratios at the University of South Carolina. Samples and standards were introduced by a Teflon PFA nebulizer and an (ESI) Apex-Q desolvating system at 150 μl min^−1^, with an Al ‘X’ skimmer cone. High-resolution mode resolved Fe from polyatomic interferences (ArN^+^, ArO^+^ and ArOH^+^). Signal intensity was measured for atomic masses 53, 54, 56, 57, 58, 60 and 61, with ^53^Cr and ^60^Ni used to correct for isobaric interferences on ^54^Fe and ^58^Fe, respectively. Signal intensity was measured over 50 cycles of 4.2 s. The first 12 cycles were discarded due to uptake and stabilization time. Any cycles with ratios more than 3 s.d. of the remaining 38 cycles were also discarded. Memory effects were minimized by a 3-min rinse (0.32 M Q-HNO_3_) between analyses. All sample intensities were blank-corrected with the mean of 38 cycles from periodic 0.1 M HNO_3_ analyses. Fe isotope ratios were calculated using a double-spike data-reduction scheme based on the iterative approach of Siebert *et al.*^57^, and are expressed relative to IRRM-14 using standard delta notation (δ^56^Fe):


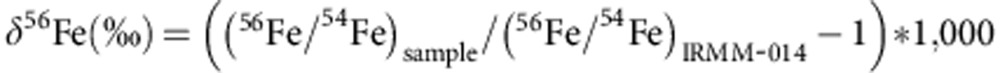


Sample ratios are expressed relative to the average of IRMM-14 standards mixed with the ^57^Fe–^58^Fe double spike in equivalent proportions and concentrations as samples. Standard-spike ratios and concentrations were assessed for deviation in IRRM-14 determination but none was found. Each sample was analysed twice, and the average is shown (Table 1). Uncertainty for δ^56^Fe is expressed as the mean s.e. of the isotope ratio over each 160 s analysis, based on previous demonstration that uncertainty of Fe isotopic measurement of a natural sample by double-spike MC ICP-MS is dominated by internal error^56^.

### Pore water O_2_ profiling

Unisense equipment was used to for shipboard O_2_ determination in surface sediments as previously described^34^. Linear calibrations were performed between aerated seawater and anoxic (N_2_ saturated) seawater before each use, with a detection limit <0.3 μmol l^−1^. A micromanipulator and SensorTracePro software controlled down-core profiling at 100 μm depth intervals. Data were converted to dissolved O_2_ concentration using empirical constraints for O_2_ saturation in seawater. Pore water profiles are surface-normalized to bottom water O_2_ determinations from shipboard Winkler titrations.

### Pore water NO_3_^−^ and NO_2_^−^ determination

Pore water samples (2 ml) were diluted with 18.2 MΩ de-ionized water to a volume of 30 ml. A 5-channel Bran and Luebbe AAIII segmented flow colorimetric autoanalyser was used to determine NO_3_^−^ and NO_2_^−^ concentration using standard analytical techniques at sea^58^. Data presented are combined NO_3_^−^+NO_2_^−^, with an analytical uncertainty of ±0.2 μmol l^−1^. Accuracy was verified by determination of nutrient standards (Ocean Scientific International) within 5% of certified values.

### Sediment mineralogical description

Polarized light microscopy, XRD and SEM with EDS assessed bulk sample mineralogy. A Philips X'Pert pro instrument with Cu-Kα radiation performed XRD. Elemental composition of targeted mineral grains was assessed from EDS generated from a Princeton Gamma Tech (IMIX-PTS) X-ray beam with a 2–3 μm diameter connected to an LEO 1450VP SEM operated at 15 keV.

### Sediment organic C determination

Total organic carbon concentrations were calculated from the difference between coulometric determination (UIC 5012 Coulometer) of total carbon (TC) and total inorganic carbon (TIC) content of dry homogenized sediments. TC was calculated from CO_2_ released during sample combustion, and TIC was calculated from CO_2_ released during heated sample reaction with 1.5 M H_3_PO_4_. Accuracy of TC and TIC determinations was assessed with anhydrous CaCO_3_ powder, with a mean recovery of 100.4±0.8% (1 s.d., *n*=15). The limit of detection (3 s.d. of blanks) was <10 μg C, equivalent to <0.03 wt% total organic carbon.

## Author contributions

W.B.H. and R.A.M. jointly conceived this study. W.B.H. designed and conducted the approach to sampling and performed all analyses, with the exception of the method for Fe isotope purification and analysis designed and assisted by S.G.J. and T.M.C. The manuscript was written and edited by W.B.H., with intellectual contributions throughout from R.A.M., S.G.J. and T.M.C.

## Additional information

**How to cite this article:** Homoky, W. B. *et al.* Distinct iron isotopic signatures and supply from marine sediment dissolution. *Nat. Commun.* 4:2143 doi: 10.1038/ncomms3143 (2013).

## Figures and Tables

**Table 1:** Summary of results from Cape margin pore water and sediment samples.

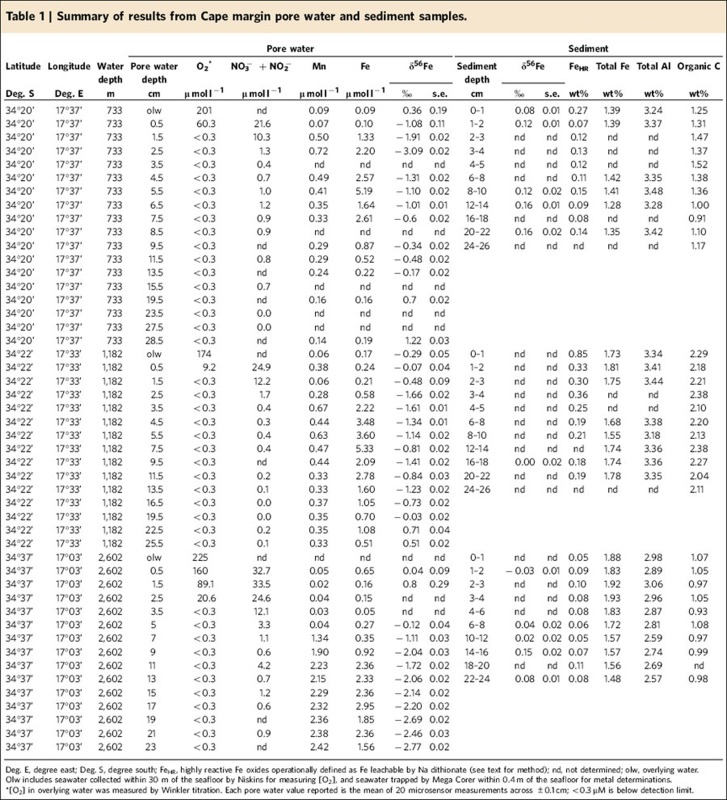																

**Table 2:** Measured elemental abundance of certified reference materials.

**CRM**	**Measured (±1 s.d.)**			**Certified (±1 s.d.)**		
	**Fe**	**Mn**	**Al**	**Fe**	**Mn**	**Al**
SLRS-5* (*n*=5, p.p.b.)	93.4±1.6	4.53±0.10	51.8±1.9	91.2±5.8	4.33±0.18	49.5±5.0
SCO-1† (*n*=1, wt%)	3.76	nd	7.15	3.59±0.13	nd	7.23±0.22
SGR-1† (*n*=1, wt.%)	2.04	nd	3.17	2.12±0.10	nd	3.34±0.11
BCSS-1* (*n*=1, wt%)	3.20	nd	6.07	3.29±0.10	nd	6.26±0.22

nd, not determined; p.p.b., parts per billion.

^*^National Research Council Canada (SLRS-5, river water; BCSS-1, marine sediment).

^†^United States Geological Survey (SCO-1, Cody Shale; SGR-1, Green River Shale).
